# Velocity-Free Acoustic Emission Source Localization for Complex Structures Using Any-Angle Pathfinding Algorithm

**DOI:** 10.3390/s26113599

**Published:** 2026-06-05

**Authors:** Dexian Li, Longjun Dong, Xuemei Wang, Guoxiang Cheng, Longbin Yang, Weikang Zhu

**Affiliations:** 1 School of Resources and Safety Engineering, Central South University, Changsha 410083, China; 255502046@csu.edu.cn (D.L.); lj.dong@csu.edu.cn (L.D.); wangxm@csu.edu.cn (X.W.); 2 State Key Laboratory of Ni & Co Associated Minerals Resources Development and Comprehensive Utilization, Jinchang 737100, China; and777@126.com (G.C.); j1733274193@163.com (W.Z.)

**Keywords:** acoustic emission sensor, acoustic signal processing, data analysis, source localization, complex structure

## Abstract

Accurate acoustic emission (AE) source localization in complex structures remains challenging due to non-straight wave propagation paths and the difficulty of obtaining reliable wave velocity models. To address these issues, this study proposes a velocity-free AE source localization method based on an any-angle pathfinding algorithm. The method integrates the Anya algorithm to estimate geometrically optimal propagation paths and a velocity-free objective function formulated under a weak anisotropy assumption. By avoiding the directional limitations of conventional grid-based pathfinding, the proposed approach provides more accurate distance estimates between potential sources and sensors without requiring prior velocity information. The effectiveness of the method was validated through an AE pulse experiment conducted on a granite specimen containing multiple cylindrical holes. The results demonstrate that the average location error of the proposed method is 19.02 mm, which is less than 38.14 mm of the traditional method and 22.90 mm of the velocity-free method using a grid-based search algorithm. Therefore, the proposed framework is applicable to two-dimensional structures or extruded three-dimensional structures, offering a feasible approach for AE source localization in complex environments.

## 1. Introduction

Acoustic emission (AE) technology, as a passive elastic wave monitoring method, has been widely applied in the spatial evolution of fractures [1,2], source mechanism investigation [3,4], and structural health monitoring [5,6]. With the continuous increase in engineering depth and structural complexity in underground excavations, deep mining, and large-scale infrastructures, the mechanical environment of materials becomes highly heterogeneous, anisotropic, and damage-prone. In such conditions, the ability to accurately locate AE sources plays a crucial role in revealing crack initiation, propagation, and coalescence processes, as well as in providing early warning information for potential instability and failure. Therefore, the reliability of AE-based analysis is strongly dependent on the accuracy and robustness of AE source localization.

The first source localization algorithm was proposed by Geiger, called Geiger’s method [7]. Thanks to the development of computer technology, some computer programs have been proposed for determining hypocenters of local earthquakes, such as HYPO71 [8] using a multiple stepwise regression method, HYPOINVERSE [9] using a generalized inverse method, HYPOCENTER [10] using a damped least-squares solution, and QUAKE3D [11] using finite-difference travel times and grid searching. These methods are generally based on pre-defined velocity models and iterative optimization, and their performance is highly sensitive to the accuracy of wave velocity and initial parameter selection. Different from Geiger’s method and its variants, joint inversion methods [12,13] calculate the source coordinates using multiple events. Relative localization methods [14,15], developed from joint inversion methods, consider that the sources close to each other would propagate in the same way. Hence, the P-wave velocity structure between the sources and the distance between each source and sensor determine the time difference in P-wave arrivals (TDOA).

Although the above methods have been successfully applied in many scenarios, their accuracy is fundamentally limited by uncertainties in wave velocity. In practical engineering applications, wave velocity is often spatially variable and affected by stress state, damage degree, temperature, and material heterogeneity. As a result, inaccurate or oversimplified velocity models can introduce systematic location errors, especially in complex structures. To remove the influence of inaccurate pre-measured wave velocity, Dong and Li [16] have developed a series of velocity-free localization methods that include two-dimensional (2-D) and three-dimensional (3-D) source localization in both simple and complex structures since 2011. Zhou et al. jointly estimated AE source and wave velocity using the constrained least squares method [17]. In 2-D cases, dispersion curves describe wave velocity as a function of frequency [18]. The peak frequency of each signal then determines its P-wave velocity. Considering that dispersion curves may change with environmental conditions, Harley and Moura [19] developed two methods using the sparse structure of the Lamb wave’s frequency-wavenumber representation. One of the methods requires no calibration data. Exploiting other information about the waveform, for example, phase and amplitude information [20,21], is also a solution to reduce the influence.

In complex structures, however, wave propagation paths are generally non-straight due to refraction, reflection, and diffraction effects caused by geometric irregularities and material heterogeneity. Under such circumstances, the assumption of straight-line propagation becomes invalid, and travel-time-based localization methods may suffer from significant errors if the actual propagation path is not properly considered. Therefore, the FastWay method [22] is proposed for the localization of AE sources in complex propagation media, in which pathfinding algorithms are introduced to calculate the distance between sources and sensors. Based on this idea, source localization methods for complex structures using an improved A* search algorithm and a velocity-free framework were developed in both 2-D [23] and 3-D cases [24,25]. However, in these methods, the calculated path length may still deviate from the true wave propagation distance due to limited search directions and grid constraints, which restrict further improvement in localization accuracy. Some researchers also developed the localization method without prior information, such as methods based on machine learning [26,27] and Delta-T [28,29,30]. Nevertheless, acquiring sufficient training data or activating artificial sources is often time-consuming and difficult in practical engineering environments.

In summary, existing AE source localization methods for complex structures still face several challenges: (1) strong dependence on P-wave velocity models or assumptions; (2) limited accuracy in complex geometries due to constrained path-search strategies; and (3) high cost or impracticality of data acquisition for data-driven methods.

In this work, we propose a velocity-free source localization method using an any-angle pathfinding algorithm (VFAA) to improve location accuracy in complex structures. The proposed method focuses on two key issues in AE source localization for complex structures: non-straight propagation paths caused by geometric obstacles and localization errors caused by uncertain P-wave velocity. The any-angle pathfinding algorithm adopted in this study is Anya [31], which allows more flexible and realistic path estimation compared with traditional grid-based approaches. Different from conventional objective functions, an improved velocity-free objective function is further developed based on a weak anisotropy assumption, aiming to enhance robustness and accuracy under complex propagation conditions.

## 2. Materials and Methods

### 2.1. Velocity-Free Source Localization Method

**Figure 1:**
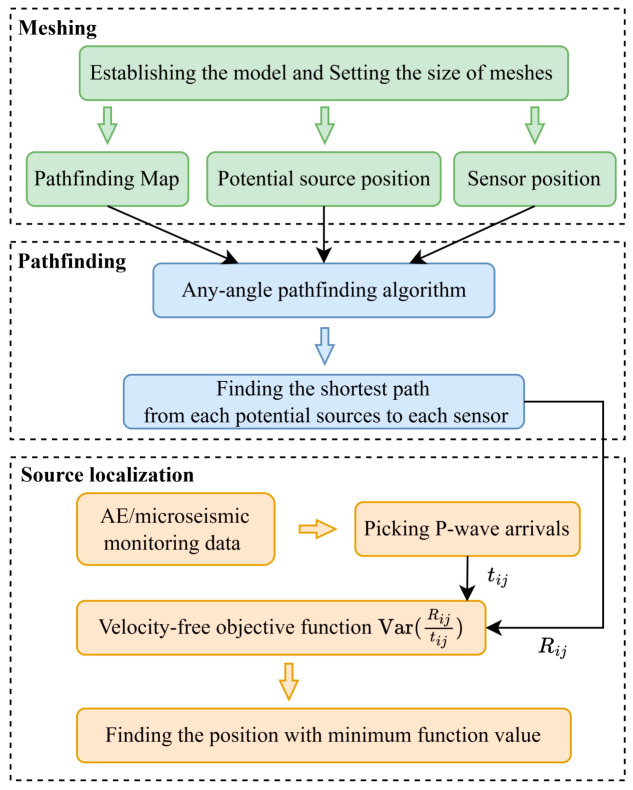
Flowchart of VFAA; $t_{ij}$ indicates the P-wave arrival of the i-th sensor minus that of the j-th sensor; $R_{ij}$ indicates the distance between the potential source and the i-th sensor minus the distance between the potential source and j-th sensor; i,j=2,3…,m, where m is the total number of sensors.

The main contribution of the proposed VFAA is the integration of the any-angle pathfinding algorithm into a velocity-free AE source localization framework for complex hole-containing structures. In this framework, Anya is used to obtain more accurate geometrical path lengths than conventional grid-constrained pathfinding, and these path lengths are then incorporated into the velocity-free objective function based on P-wave arrival-time differences. This coupling reduces grid-induced path-length errors while avoiding the need for premeasured P-wave velocity. The VFAA consists of three parts: meshing the model, finding the shortest path from each potential source to each sensor, and then getting the source position according to the velocity-free objective function. Figure 1 illustrates the flowchart for VFAA. In this section, we will detail each part.

#### 2.1.1. Meshing the Model

When meshing the model, we must determine the appropriate mesh size, considering both location accuracy and time consumption. After that, prepare the pathfinding map and coordinates of start points (e.g., potential sources) and target points (e.g., sensors) for the any-angle pathfinding algorithm. In the map, we use two flags to distinguish traversable and non-traversable cells.

#### 2.1.2. Anya: An Optimal Any-Angle Pathfinding Algorithm

**Figure 2:**
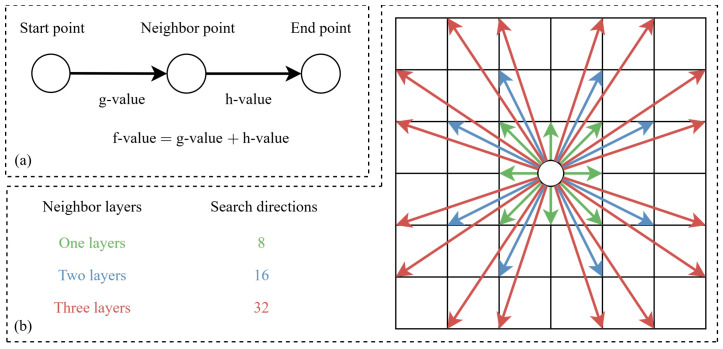
Illustration of the A* algorithm. (**a**) Evaluation mechanism of a neighbor node in the A* algorithm; (**b**) Search directions obtained by expanding neighbor layers in grid-based search.

In this section, we introduce the framework of the Anya algorithm and make some comparisons with the A* algorithm. Figure 2a illustrates the evaluation mechanism of a neighbor point in the A* algorithm. For a given neighbor point, the evaluation value, denoted as the f-value, is defined as the sum of the accumulated path cost from the start point (g-value) and the heuristic estimation from the neighbor point to the target (h-value). During the search process, the neighbor point with the minimum f-value is selected as the next node to be expanded. This strategy ensures the optimality of A* under the condition that the heuristic function is admissible.

However, it should be noted that the A* algorithm performs the search on discrete grid points, and the possible propagation directions are inherently restricted by the predefined neighborhood structure. As illustrated in Figure 2b, when only the first neighbor layer is considered, the number of available search directions is limited to eight. To approximate wave propagation along arbitrary angles, additional neighbor layers must be introduced, which increases the number of search directions to 16 or 32. Although expanding the neighbor layers improves the angular resolution of the search, it inevitably leads to a rapid growth in computational complexity. Specifically, the number of neighbor points to be evaluated increases exponentially with the number of layers, significantly enlarging the open list and causing repeated node expansions. Therefore, the trade-off between angular resolution and computational efficiency represents a fundamental bottleneck of grid-based pathfinding algorithms in complex AE propagation environments. Even with an increased number of neighbor layers, the resulting paths remain constrained by the underlying grid discretization, producing piecewise linear and zigzag trajectories instead of true shortest paths. Consequently, conventional grid-based A* algorithms are fundamentally incapable of achieving efficient and accurate any-angle pathfinding, which motivates the introduction of interval-based search strategies such as the Anya algorithm.

**Figure 3:**
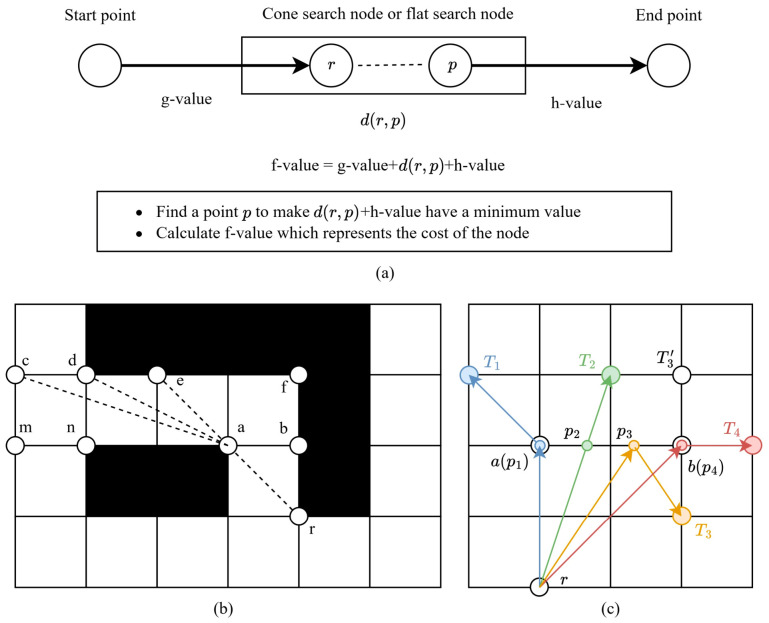
Illustration of the Anya algorithm. (**a**) Evaluation of the f-value for a search node in the Anya algorithm; (**b**) Successor generation for an Anya node, including cone search nodes and flat search nodes; (**c**) Geometric illustration of selecting the optimal point within an interval by projection to minimize the heuristic cost.

Instead of A* searching over grid points, Anya searches over intervals, which are sets of continuous points. All points in an internal have the same y coordinate, allowing a single search node to implicitly represent an infinite number of potential propagation directions. In this way, Anya fundamentally overcomes the angular discretization limitation inherent in grid-based pathfinding algorithms. A search node in Anya is represented by (I,r), where I denotes an interval and r is the root point from which all points in I are visible. Because the node is defined by a point–interval pair rather than a single grid point, the cost evaluation must consider the distance between r and any point p∈I. As illustrated in Figure 3a, Anya adopts a heuristic formulation analogous to that of A* but extended to continuous domains. For a node (I,r), the evaluation function is defined as

(1) $$f\left(n\right)=\underset{p\in I}{\min}\left[g\left(r\right)+d\left(r,p\right)+h(p)\right],$$

where g(r) is the accumulated path length from the start point to the root r, d(r, p) is the Euclidean distance between r and a candidate point p within the interval, and h(p) is the heuristic estimate from p to the target. The successor generation mechanism of Anya is illustrated in Figure 3b. For a node ([a, b], r), two types of successor nodes are generated: cone search nodes and flat search nodes. Cone search nodes correspond to cases where the visible region changes due to obstacles, resulting in new intervals such as ([e, f], r), ([d, e], a), and ([c, d], a). In contrast, flat search nodes represent the continuation of visibility along the same direction, producing successors such as ([n, a), a) and ([m, n], a). The combination of cone and flat nodes ensures both completeness and optimality of the search, while maintaining the any-angle property. The minimization over p is not performed by exhaustive enumeration but by geometric projection, which allows the optimal point p to be determined efficiently in continuous space, as shown in Figure 3c.

Through this interval-based representation and projection-based cost evaluation, Anya achieves true any-angle pathfinding without sacrificing optimality or incurring the combinatorial explosion associated with multi-layer neighbor expansion in conventional A* algorithms.

Anya is a two-dimensional pathfinding algorithm, so we can use it to find the shortest path in any two-dimensional model. For some special cases, we can calculate the length of the shortest path in a three-dimensional model using Anya and the Pythagorean theorem, as shown in

(2) $$L_{i}=\sqrt{{\left(L_{i}^{xy}\right)}^{2}+{\left(i_{z}-s_{z}\right)}^{2}},$$

where $L_{ij}$ is the length of the shortest path from point $i(i_{x},i_{y},i_{z})$ to $s(s_{x},s_{y},s_{z})$; $L_{i}^{xy}$ the length of the shortest path from $\left(i_{x},i_{y}\right)$ to $\left(s_{x},s_{y}\right)$.

#### 2.1.3. Velocity-Free Objective Function

The objective function judges whether a potential source is close to the real source. Firstly, we define the time difference in P-wave arrivals $t_{ij}$ and distance difference $R_{ij}$ in Equation (3). The traditional method uses Equation (4) as an objective function with constant velocity, where *c* is the constant P-wave velocity. To remove the influence of velocity deviation, Equation (5) assumes that P-wave velocity may vary with time but should be uniform in space (i.e., isotropic). In Equation (5), the parameter v is the unknown P-wave velocity and can be calculated by iterations. By using Equation (6), we assume that P-wave velocity is not uniform in terms of time and space but should have a minimum variance from the real source to sensors. Theoretically, methods using Equation (6) would obtain better results compared with those using Equation (5) when the monitoring area has weak anisotropy. In some cases, $R_{i1}$ would be equal to 0, which increases the variance of velocity. To fully utilize all available data and avoid cases where $R_{i1}=0$, Equation (7) is formulated using pairwise distance differences and arrival-time differences among all sensors. In this formulation, the equivalent velocity set of a candidate source location is defined as $\hat{v}=\left\{\frac{R_{jk}}{t_{jk}}|R_{jk}\neq 0,\;j,k=2,\ldots ,m\right\}$. In Equations (6) and (7), $D\left(\cdot \right)$ calculates the variance of a set.

(3) $$\begin{array}{c} t_{ij}=t_{i}-t_{j},\;R_{ij}=L_{i}-L_{j}\\ i,j=1,2,\ldots ,m \end{array}$$

(4) $$\min F\left(x,y,z;c\right)=\sum_{i=1}^{m}{\left(t_{i1}-\frac{R_{i1}}{c}\right)}^{2}$$

(5) $$\min F\left(x,y,z,v\right)=\sum_{i=1}^{m}{\left(t_{i1}-\frac{R_{i1}}{v}\right)}^{2}$$

(6) $$\min F\left(x,y,z\right)=D\left(\left\{\frac{R_{i1}}{t_{i1}}|i=2,\ldots ,m\right\}\right)$$

(7) $$\min F\left(x,y,z\right)=D\left(\hat{v}\right)$$

Considering that the mean equivalent velocity varies among different candidate source locations, directly using the variance as the error function may be affected by the magnitude of the velocity, making it difficult to fairly compare the dispersion of equivalent velocities at different candidate locations. Therefore, an improved objective function is proposed, as shown in

(8) $$\min F\left(x,y,z\right)=\sqrt{D\left(\hat{v}\right)}/E(\hat{v}),$$

where E(·) calculates the mean value of a set. In this formulation, the coefficient of variation is adopted as the objective function to reduce the influence of velocity magnitude.

### 2.2. Experimental Setup

#### 2.2.1. Experiment Configuration

**Figure 4:**
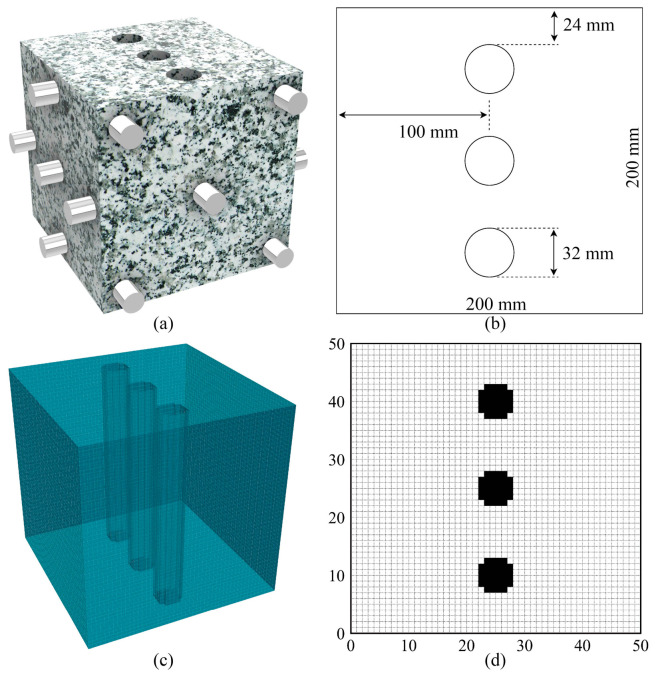
Experimental setup: (**a**) rock sample and sensor position; (**b**) diameter and position of cylindrical holes; (**c**) meshing the three-dimensional model; (**d**) illustration of a pathfinding map where black cells are untraversable.

**Figure 5:**
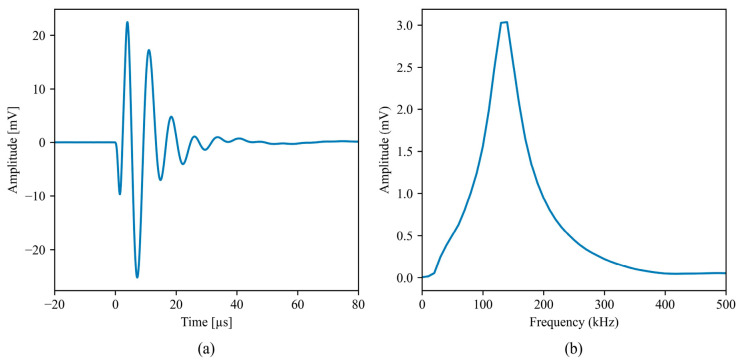
Characteristics of the pulse source signal: (**a**) time-domain waveform and (**b**) amplitude spectrum.

An AE experiment was conducted on a granite specimen with dimensions of 200 mm × 200 mm × 200 mm to validate the proposed VFAA. As shown in Figure 4a, the specimen was instrumented with 20 VS45-H piezoelectric sensors (Vallen Systeme GmbH, Wolfratshausen, Germany) mounted on the four lateral surfaces to ensure adequate spatial coverage. Table 1 shows the coordinates of sensors. AE signals were acquired using an AMSY-6 data acquisition system (Vallen Systeme GmbH, Wolfratshausen, Germany). The sensors have a nominal frequency response ranging from 20 kHz to 450 kHz. The preamplifier gain, sampling rate, detection threshold, and pre-trigger sampling points were set to 34 dB, 10 MHz, 30 dB, and 1000, respectively. Three vertical cylindrical holes were drilled through the specimen, as illustrated in Figure 4b. The holes were deliberately introduced to create a complex internal structure, forcing wave propagation paths to deviate from straight lines and thereby providing a challenging test scenario for pathfinding-based localization methods. Such configurations are representative of engineering structures containing voids, boreholes, or embedded components. During the experiment, each sensor sequentially acted as an active pulse source and emitted four excitation signals, while the remaining sensors recorded the waveforms in real time. The same excitation setting was used for all active sensors to ensure consistency among different source positions. The Vallen system provides two pulse settings, namely the Normal setting and the Low setting, with different ranges of centroid frequencies. In this experiment, we adopted the Normal setting with a centroid frequency of 140 kHz. Figure 5 shows the time-domain waveform and amplitude spectrum of the emitted source signal. The known locations of the pulse sources provide reference positions for quantitatively evaluating the localization accuracy of different algorithms under identical experimental conditions.

#### 2.2.2. Pathfinding Configuration

**Figure 6:**
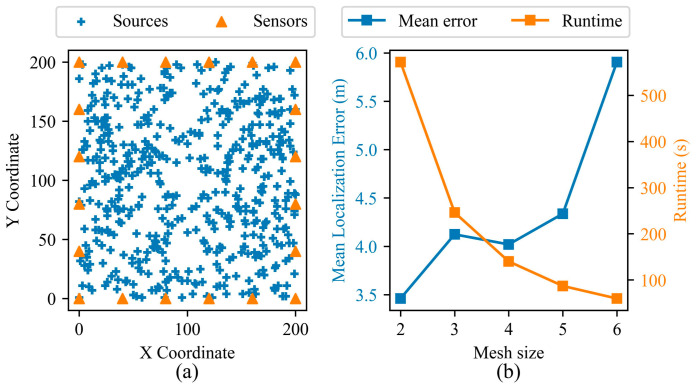
(**a**) Randomly generated source locations. (**b**) Influence of mesh sizes on localization accuracy and runtime for VFAA.

Mesh size has a significant impact on the positioning accuracy and computation time of the localization algorithm. To select a suitable grid size, we designed a numerical simulation using 500 randomly selected numerical events and 20 sensors (See Figure 6a). The travel times were generated using the fine 1 × 1 m mesh for the model shown in Figure 4b. In the simulation, the P-wave velocity was set to 3000 m/s, and the acquisition sampling rate was set to 10 kHz. Uniformly distributed arrival-time noise within $\left[-1,1\right]$ ms was added to the generated travel times to simulate practical engineering conditions. After that, the source location was conducted using the Anya algorithm with meshes from 2 × 2 m to 6 × 6 m.

Figure 6b demonstrates the mean localization error and runtime of the proposed velocity-free any-angle pathfinding algorithm (VFAA). Overall, finer meshes can improve localization accuracy but require more computational time. In contrast, coarser meshes can reduce computational cost and improve efficiency, but they also lead to larger localization errors. A slight exception is observed for the 3 × 3 m mesh, where the localization error is larger than that for the 4 × 4 mm mesh. This is because the 200 × 200 m model domain cannot be evenly divided by a 3 m mesh, and the sensor coordinates cannot be exactly represented on this mesh. The resulting coordinate discretization error affects the calculated travel times, thereby increasing the localization error.

Therefore, the specimen was discretized into uniform cubic meshes with a resolution of 4 × 4 × 4 mm to construct the computational model for pathfinding, as shown in Figure 4c. The cylindrical holes were mapped onto the grid as non-traversable regions, resulting in a pathfinding map illustrated in Figure 4d, where black cells indicate obstacles and white cells represent homogeneous granite.

## 3. Results and Discussion

### 3.1. Shortest Path Comparison Between A* and Anya Algorithms

**Figure 7:**
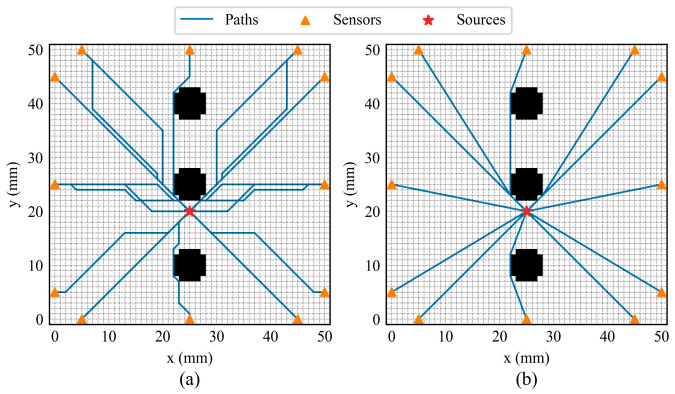
Example of pathfinding results using (**a**) the A* algorithm and (**b**) the Anya algorithm. The star indicates the starting point, while the triangle represents the target point. The black blocks indicate the obstacles.

Figure 7 demonstrates the example of searching for the shortest path from the start point (star) to target points (triangles) using the A* algorithm (Figure 7a) and the Anya algorithm (Figure 7b). As shown in Figure 7a, the A* algorithm produces zigzag paths constrained by discrete search directions, particularly in regions near obstacles. These artificial turns introduce systematic overestimation of propagation distances, even when the free space allows a straight-line path. In contrast, Figure 7b demonstrates that the Anya algorithm generates smooth and nearly straight paths in unobstructed regions, while naturally bending around obstacles along visibility boundaries. The resulting paths exhibit strong geometric symmetry and closely approximate the true Euclidean shortest paths between the source and sensors.

For acoustic emission source localization, such differences in path geometry are critical. Errors in path length estimation directly propagate into the calculation of TDOA, leading to biased source location results. By eliminating grid-induced directional errors, the Anya-based approach provides more accurate distance estimates, which significantly improve the robustness and precision of velocity-free localization in complex structures.

### 3.2. Influence of Sensor Number on Localization Accuracy

**Figure 8:**
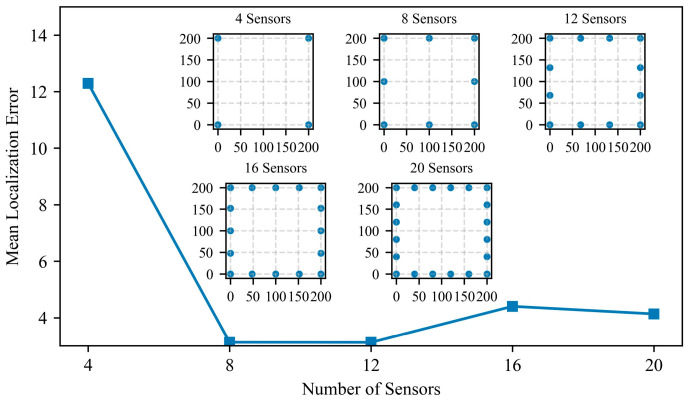
Mean localization errors under different sensor numbers and corresponding sensor layouts.

To investigate the influence of sensor number on localization accuracy, five sensor networks containing 4, 8, 12, 16, and 20 sensors were designed, respectively. The settings of the simulation were the same as those described in Section 2.2.2. The sensor layouts and mean localization errors are shown in Figure 8. The mean localization errors of five sensor layouts were 12.29, 3.13, 3.12, 4.41, and 4.14 m, respectively. A clear improvement can be observed when the number of sensors increases from 4 to 8, indicating that the four-sensor network provides insufficient geometric constraints for stable source localization. After 8 sensors, however, the mean error does not decrease monotonically. The 8- and 12-sensor networks show comparable performance, while the 16- and 20-sensor networks produce slightly larger mean errors. This suggests that localization accuracy is not controlled solely by the number of sensors, but also by the spatial configuration of the network and the stability of the objective function. Additional sensors may introduce sensor pairs with small arrival-time differences or weak geometric constraints, which can amplify the effect of arrival-time perturbations in variance-based velocity formulations. Therefore, increasing the number of sensors does not necessarily guarantee lower localization error unless the added sensors improve the overall geometric coverage and provide independent constraints on the source location.

### 3.3. Location Results

**Figure 9:**
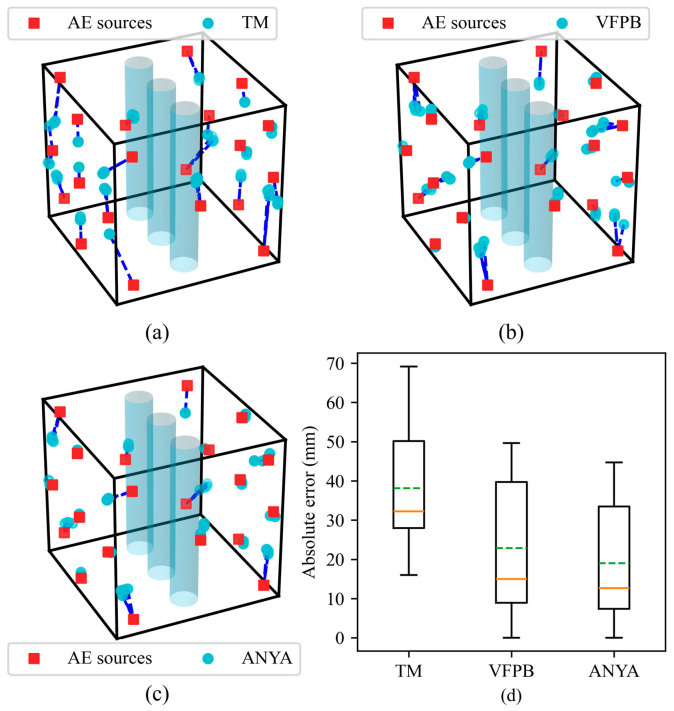
Location results of (**a**) TM, (**b**) VFGB, and (**c**) VFAA; (**d**) absolute location errors of three methods; dash lines and solid lines indicate the average and median, respectively.

The source localization results obtained using three different methods are presented in Figure 9a–c, including the traditional method based on the isotropic velocity assumption (TM), VFGB, and VFAA. In these graphs, squares denote the true AE source positions, while circles indicate the corresponding estimated locations.

As shown in Figure 9a, the TM exhibits relatively large deviations between the true and estimated source positions. The localization errors tend to increase in regions where wave propagation paths are strongly distorted by geometric obstacles, reflecting the sensitivity of isotropic velocity assumptions to complex propagation environments. Although VFGB (Figure 9b) partially mitigates these errors by incorporating a pathfinding algorithm and velocity-free constraints, noticeable deviations remain due to grid-induced path discretization.

In contrast, Figure 9c demonstrates that the proposed VFAA produces estimated source locations that are consistently closer to the true positions across the entire specimen. The displacement vectors between the true and estimated locations are generally shorter and exhibit less directional bias, indicating a more accurate and stable reconstruction of propagation distances in the presence of internal obstacles.

A quantitative comparison of localization accuracy is provided in Figure 9d, which presents boxplots of the absolute localization errors for the three methods. The average localization errors of TM, the velocity-free method combined with a grid-based pathfinding algorithm (VFGB), and VFAA are 38.14 mm, 22.90 mm, and 19.02 mm, respectively, while the corresponding median errors are 32.25 mm, 14.97 mm, and 12.65 mm. These results confirm that accurate estimation of propagation path lengths plays a critical role in velocity-free AE source localization. By eliminating grid-induced directional errors and providing geometrically optimal shortest paths, the Anya-based pathfinding strategy enables more reliable distance estimation, which directly translates into improved localization accuracy in complex structures.

## 4. Conclusions

This work develops a velocity-free acoustic emission source localization method suitable for complex structures by integrating an any-angle pathfinding strategy with a weakly anisotropic objective function. By employing the Anya algorithm, the proposed method overcomes the inherent directional limitations of conventional grid-based searches, such as the 8-direction constraint in two-dimensional models and the 26-direction constraint in three-dimensional cases, and consistently yields geometrically optimal shortest propagation paths.

The improved path estimation directly enhances the accuracy of distance differences between the source and sensors, leading to calculated time differences in P-wave arrivals (TDOAs) that are more consistent with the observed signals. In addition, the velocity-free objective function formulated under a weak anisotropy assumption effectively mitigates the influence of spatial and temporal velocity variations, further improving the robustness of source localization in heterogeneous propagation environments.

Experimental validation using an AE pulse test on a granite specimen with internal cylindrical holes demonstrates the effectiveness of the proposed approach. Compared with the traditional isotropic method and the velocity-free method based on grid-based pathfinding, the proposed VFAA reduces the average localization error by approximately 50.14% and 16.94%, respectively. Beyond the reduction in mean error, the VFAA also exhibits improved stability, as reflected by lower median errors and a more compact error distribution.

Owing to its reliance on geometric path optimization rather than predefined velocity models, the VFAA is well suited for practical applications involving complex internal structures, such as rock masses containing voids or boreholes, engineered components with embedded features, and other heterogeneous media where accurate velocity characterization is challenging. Despite these advantages, several limitations remain in the current implementation. The proposed framework is applicable to two-dimensional structures and extruded three-dimensional structures. In addition, the Anya algorithm adopted in this study requires a structured pathfinding map as its input representation. Future work will focus on extending the VFAA framework to fully three-dimensional pathfinding and more flexible spatial discretization schemes, further enhancing its applicability to more general engineering scenarios.

## Figures and Tables

**Table 1:** Coordinates of sensors (unit: mm).

NO.	X	Y	Z
1	20	0	180
2	180	0	180
3	180	0	20
4	20	0	20
5	100	0	100
6	200	100	180
7	200	180	100
8	200	100	20
9	200	20	100
10	200	100	100
11	180	200	180
12	20	200	180
13	20	200	20
14	180	200	20
15	100	200	100
16	0	100	180
17	0	20	100
18	0	100	20
19	0	180	100
20	0	100	100

## Data Availability

Data is contained within the article.
